# A thematic analysis assessing clinical decision-making in antipsychotic prescribing for schizophrenia

**DOI:** 10.1186/s12888-018-1872-y

**Published:** 2018-09-10

**Authors:** Rossela Roberts, Abigail Neasham, Chania Lambrinudi, Afshan Khan

**Affiliations:** 1 Betsi Cadwaladr University Health Board, Bangor, UK; 2 Cardiff University, Cardiff, UK

**Keywords:** Antipsychotics, Schizophrenia, Guidelines, Qualitative, Evidence based medicine

## Abstract

**Background:**

In recent decades atypical antipsychotics have increased treatment options available for schizophrenia, however there is conflicting evidence concerning the trade-off between clinical efficacy and side effects for the different classes of antipsychotics. There has been a consistent increase in atypical antipsychotic prescribing compared to typical, despite evidence showing that neither class is superior. This leads to the question of whether prescribers are selective in their uptake of research evidence and clinical guidelines and if so, what influences their choice.. This study aims to identify the factors that contribute to the prescribing choice and how these can be used to aid knowledge translation and guideline implementation.

**Methods:**

A thematic analysis study was conducted using data from 11 semi-structured interviews with clinicians with experience in prescribing for schizophrenia.

**Results:**

The analysis identified five themes underpinning prescribing behaviour**:** (1) ownership and collaboration; (2) compromise; (3) patient involvement; (4) integrating research evidence; and (5) experience.

**Conclusion:**

The themes mapped to various degrees onto current models of evidence-based decision making and suggest that there is scope to re-think the guideline implementation frameworks to incorporate recurring themes salient to clinicians who ultimately use the guidelines. This will further translation of future evidence into clinical practice, accelerating clinical progress.

## Background

NICE Clinical Guideline 82 [1] and the subsequent Clinical Guideline 178 “Psychosis and schizophrenia in adults: prevention and management” [2] replacing the initial NICE Clinical Guideline 1 “Schizophrenia: Core Interventions in the Treatment and Management of Schizophrenia in Primary and Secondary Care” [3] firmly advise that no preference should be given to typical or atypical antipsychotics in the treatment and management of schizophrenia.

However, research suggests that atypical antipsychotics remain the prevalent choice of medication: Marston et al. [4] found that atypical antipsychotics accounted for 66.7% of the total antipsychotics prescribing, and the Prescription Cost Analysis data shows a continuous decrease in typical and increase in atypical antipsychotic prescriptions between 2006 and 2014 [5].

This paper aims to understand why atypical antipsychotics continue to be the ubiquitous prescribing practice despite robust research evidence indicating no clinical efficacy advantages (apart from clozapine) [6–9]. While many trials do not report a difference in clinical efficacy there is a well-recognised difference in side effects between typical and atypical antipsychotics [9]. Typical antipsychotics are associated with extrapyramidal side effects, whereas atypical antipsychotics are associated with metabolic side effects [6, 10]. It has been found that these side effects are dose related [9]. Side effects have been found to be an important factor in prescribers decision making [11], which could explain the increased prescription rate of atypical antipsychotics as there side effects are viewed as more favourable and the avoidance of extrapyramidal side effects may have encouraged more prescribers to use atypical antipsychotics [12].

Although the published literature [11, 13, 14] offers insight into clinical decision making, an exploration of how the contributing factors map onto the evidence-based medicine (EBM) models was needed.

Clinical decision-making is informed by the principles of EBM. A recent model of evidence based decision-making states that there should be a balance between four main factors: research evidence; patient’s preferences; the clinical scenario and the clinical environment. Clinical expertise is needed to tie these four parts together and assess the best treatment options for the patient [15].

Banning [13] found that inexperienced practitioners rely more on guidelines than those with experience, in whom decision-making becomes more intuitive. According to Bate [14], information is processed using two independent cognitive systems. System one is described as ‘an intuitive, fast, automatic and effortless process,’ in contrast to system two which is described as ‘careful, rational analysis and evaluation of available information’. The development of system one depends on experience and clinical teaching, whereas system two relies on the insight into the limitations of their knowledge. It is recognised that there is a four-stage conscious competence model of learning in which there is a cycle between system one and two depending on one’s awareness of their competency in a particular skill.

Recent research identifies barriers to guideline implementation, including organisation resources, characteristics of the healthcare professionals and their perception of the guidelines and treatment [16]. Recognising the barriers that prevent the delivery of optimal evidence-based care is important as an inability to adequately utilise research leads to ineffective care [17]. However, barriers to guideline implementation and knowledge translation could not account for all factors that may contribute to the prescribing trends. This article aims to explore what other factors may be involved in the decision-making in prescribing antipsychotics, by using thematic analysis to investigate clinicians’ own narrative when asked to rationalise their choice of prescribing.

## Methods

### Recruitment and participants

The study used a purposive sampling and a data saturation strategy. Eleven clinicians were recruited from a Health Board in Wales, and the inclusion criteria was limited to experience in antipsychotic prescribing for schizophrenia, and willingness to take part in the study. The sample included two nurse prescribers, three junior doctors and six consultants (two of which professors with significant experience and seniority). This heterogeneous sample meant that a wide range of views were explored. Although the sample may not be representative of the UK, this was not deemed to be an issue of significance in this exploratory, hypothesis generating qualitative study. The amount of information obtained determines the number of participants needed; as many themes as possible are produced and when no new themes emerge, data saturation has been reached and no more participants are required [18]. Recruitment of participants stopped when no new themes emerged.

### Data collection

The semi-structured interviewers conducted required participants to consider two identical case vignettes - which were designed to describe patients with presentations suggestive of schizophrenia. Participants were required to describe their clinical reasoning but were not explicitly informed that the study investigated factors that contributed to their choice of antipsychotic, to avoid leading. Participants were asked to work through the case vignettes in a ‘think-aloud’ format, a method requiring the subject to explicitly verbalise their thinking process [19]. This method was chosen as it allows greater insight into the immediate thoughts; research has shown that the think-aloud technique is one of the most valid tools for assessing higher level thought processes as well as for comparing participants performing the same task [19]. For this method to be effective, the vignette used must be complex enough to remove the possibility of the candidate completing it automatically. These interviews were recorded and transcribed verbatim.

### Data analysis

The interviews were analysed using deductive thematic analysis, a qualitative method that results in the identification of themes throughout a data set. It allows the analysis of the commonalities between different discussions of the same topic [20]. The thematic analysis method followed the six phases identified by Braun and Clarke [21]. The coding was carried out manually and moderated to ensure their consistency and validity in relation to the data. Inter-rater reliability achieved a similarity of at least 80% (by simple percent agreement) between the researchers which suggests that the themes were reliable.

A code is a label that can be assigned to parts of the interview with similar meaning in the process of coding [22]. Each interview was coded individually using the transcribed copy of the interview and the recording. The creation of codes helped to reduce the volume of data and created a new structure for analysis. In this study, semantic codes were identified in order to provide a description of all the data. Semantic codes refer to the surface meaning of the data [20] Coding was completed using a cutting and sorting method [23]. From this process, a coding framework was generated (Appendix), which led to families of codes coalescing to form the themes presented below.

## Results

Five main themes emerged during thematic analysis of the data: (1) ownership and collaboration; (2) compromise; (3) patient involvement; (4) integrating research evidence; and (5) experience.

### Patient involvement

This theme explores firstly whether the participants consider patients in their decision-making and secondly whether this involvement appears to be tokenistic or true. According to guidelines, patients should be included in treatment decisions. The question can then be asked does a participant conscientiously following guidelines make them more likely to include a patient’s opinions.

Most participants recognised the importance of discussing treatment options and side-effects with the patients,

*“if you get a relationship right with the patient, your likelihood of success is better” (*Participant nine).

However, the ways in which participants engaged with patients varied. Participant nine states that, *“the pharmacist comes in and is usually very happy to discuss with the patient*” indicating they would not personally discuss these issues with the patient.

Other participants suggest that while they are happy to hear the patient’s views they will overrule their views if they disagree with what they feel is best.

*“I’ve got more psychiatric experience than the patient has…what I would do here, I would first try Amisulpride”* (Participant eight).

This participant did not mention guidelines at any point during their interview.

Participant ten discusses and follows NICE guidelines, describing the discussion with the patient as a “negotiation”. This is important to them as they recognise that many patients do not take the medication that they are prescribed.

Other participants disagree; while they believe that the patient should be consulted they think that too much choice could be viewed as uncertainty about antipsychotics:

*“the patient may be left with the idea that erm, we’re not one hundred percent certain, you know, which will be effective and which won’t be really*” (participant 6).

During their interview, they discuss the Maudsley guidelines but not NICE guidelines.

### Ownership and collaboration

It was found that participants took varying levels of responsibility for parts of the prescribing process depending on the aspect of care they were discussing.

#### Sub-theme 1: Taking full responsibility

Some of the participants took full responsibility for the care of the patient, as indicated by use of the first person singular. For example, participant one consistently uses *‘I’* when gathering information and forming a diagnosis. They also take ownership of the risks associated with prescribing.

“So now I can change the Olanzapine, but I can’t change the diabetes” (Participant 1).

#### Sub-theme 2: Sharing responsibility

Other participants indicated they would share responsibility of patient care, as indicated by using the first person plural. For example, when discussing risk management, participant one says “*we need to manage”.* Participant two uses “*I”* when planning the assessment and their immediate plan, however they use “*we*” when discussing antipsychotic prescribing. Some participants recognise the importance of working with people to reach a diagnosis in borderline cases.

“One of the things you gain by people being in hospital is you get the chance for a few different people to see them err, over a period of a few days, and that can be tremendously useful.” (Participant 3).

This could match the uncertainty that they feel with prescribing; in comparison to the other participants they are slow to recommend antipsychotic prescribing.

Participant nine indicated they would use other healthcare professionals to help gather information saying, ‘*you might get some information from the GP*,’ and *‘the nursing staff observe.’* However, when taking about the treatment plan, they said, *‘then you decide,’*which suggests they were ultimately the one to make the decision about treatment.

Many participants worked with the patient to inform their decisions.

‘it is important when you’re prescribing a drug to a patient, to discuss it with them, you’ve got to talk about their hopes, fears and expectations’. (Participant eight)

#### Sub-theme 3: Putting the onus on others

Participants often shifted responsibility onto other members of the team when they were talking about research.

‘*They have done trials with a lot of antipsychotics…it’s the commissioners and things like that, they say there is not a lot of evidence’* (Participant 1).

The use of the third person plural in this context suggests the participant did not feel responsible for assessing and applying research evidence to their clinical practice.

### Experience

Participants refer to their clinical experience in many ways; some frequently use the experience of previous patients to guide their clinical decisions. Others appear to have less confidence in their clinical experience. This theme questions how this disparity impacts prescribing. The varying levels of experience included in this study allows insight into what guides prescribers at different stages of their training.

Some participants place greater emphasis on clinical experience, while recognising that they themselves have little clinical experience in prescribing antipsychotics. Their clinical experience is based on what they see experienced psychiatrists do on a day-to-day basis*,*

*“as a non-medical prescriber, it would be very, very err, unwise I think to go against erm ... what erm, seems to be erm, the evidence base norm amongst experienced psychiatrists that I work with certainly.” (*participant six).

This awareness of their limited clinical experience is emphasised by their reliance on collaboration as discussed in theme four.

Other participants discuss previous patients that they have treated, their beliefs with regards to treatment and assessment appear to be based on their experience with previous patients.

*“I’ve had patients who err ... presented with anorexia and I was treating them, and then they started to develop psychosis” (participant nine)*

### Integrating research evidence

This theme explores whether the data supports a hypothesis that the knowledge participants have of current clinical guidelines and published research influences the treatment plan, or whether they rely on other factors. Throughout this theme, it was found that participants use evidence in varying ways.

*‘There’s now this thing that you can prescribe atypical or typical...the guidance was on atypicals, now it’s changed,’* (Participant one) recognising that neither group of antipsychotics is superior. However, despite this knowledge, when discussing the plan, there was no consideration of typical antipsychotics:

*‘Aririprazole would be my first choice…start with atypicals’*

Other participants recognise the importance for a prescriber to be confident about keeping up-to-date, “*I think erm, you’d have to be confident in looking at the research evidence”*. (Participant six). This statement is in contrast to their lack of confidence in their prescribing ability, where they rely on the research.

Participant four has based their belief that patients would rather have efficacious drugs with side effects than drugs with reduced efficacy on evidence. They then use research, which also follows this belief to guide their practice:

*“because patients seem to want efficacy, I look at efficacy data for antipsychotics primarily, and not just from my own study, but from a lot of very good recent meta-analyses.”*

Other participants disagree:

*“The clinical trial evidence about what they do and don’t do is close to worthless”* (participant three).

Some participants base most of their knowledge on observation and teaching sessions, without mentioning research evidence.

*“A lot of my knowledge of antipsychotics comes from a discussion I had with a Professor”.* (Participant eight)

### Compromise

This theme refers to the compromise that prescribers make between the clinical efficacy and side effects of antipsychotics. Some participants indicated that efficacy is the most important aspect of treatment.

*“Don’t do a trade-off between side effects and efficacy. What I say is that erm ... most ... most patients are willing to put up with significant side effects if the efficacy is good. And they’re not willing to put up with side effects if there is no efficacy”* (Participant four).

They base this statement on a research paper which found patients primarily want efficacy, whereas clinicians think that patients want to avoid side effects. This certainty that the choice should be made on efficacy is matched by the lack of discussion concerning risk during their interview.

Others felt that the side effect profile of the drug is more important.

“*I’d be very wary of side effects*” and “*I would urge caution really, and erm ... look at the erm, effectiveness of the ... the drugs”* (Participant six).

This caution with side effects is matched by their awareness of risk.

## Discussion

The results highlight important aspects of practitioner’s reasoning for the way in which they prescribe antipsychotics. All the themes identified in this study map onto aspects of the EBM model developed by Haynes et al. [15], providing validation of these factors. It is clear however, that participants place different weighting on each aspect.

### Patient involvement

The first theme corresponds with the importance of patient’s preferences highlighted by Haynes et al. [15]. There appears to be a strong association between patient involvement and the use of NICE guidelines. As discussed by Banning [13] those with less clinical experience tend to rely more heavily on guidelines. Therefore, this could suggest that those with less clinical experience involve the patient more than those with higher levels of clinical experience. Another explanation could be that modern medical training encourages patient involvement so those with less clinical experience are more likely to focus more heavily on patient preference. Shared decision-making should be advocated in all aspects of medicine, however it is especially important in mental health where there are many treatment options available, none of which have obvious superiority [24]. As well as the obvious ethical improvements this provides, it may have other benefits such as improved adherence to treatment [25].

### Ownership and collaboration

The gathering of clinical information is another factor discussed by Haynes et al. (2002) and this is assessed in the themes. Many participants were happy to take ownership of the information gathering process and recognised its importance in creating a treatment plan. Other participants recognise the importance of collaborating with other professionals to carry out a comprehensive assessment of the situation. This ties in with NICE guideline recommendations, which suggest that other possible organic causes should be ruled out prior to the diagnosis of schizophrenia [2]. Bradley [11] suggested that prescribers require more information for ‘high risk’ treatments; the same appears to be true for clinical scenarios. Participants who identified more risk with a clinical scenario and the wellbeing of a patient wanted more information and discussed diagnosis in further detail.

### Experience

Haynes, Devereaux and Guyatt [15] stated that clinical expertise is needed to utilise all the information to come up with a treatment plan. Many participants agree with the importance of clinical experience. Banning [13] believes that with experience, decision making becomes more innate and professionals rely less on guidelines. This suggests that professionals place great importance on what has worked for their previous patients. It is essential that they do not only look back to what has worked with previous patient, but that they also look forward to the new directions of research.

### Integrating research evidence

Haynes [15] also highlights the importance of incorporating research evidence into clinical decision making. We found that several participants use research to guide their practice, however only three participants said they would refer to guidelines. Guidelines provide recommendations for the treatment of schizophrenia and the NHS promotes their implementation. The lack of implementation highlights the important reasons for conducting this study. Identification of these factors could in the future have an impact on guideline distribution and implementation. The implementation of guidelines has been addressed by Rowlands [26] who suggest that a strong clinical lead is needed who can distribute the evidence and encourage its use. Implementation of evidence is also addressed in Roger’s Diffusion of Innovation [27], where it is suggested that the individual scenario must be identified and assessed. In the UK guidelines are released nationwide and implementation is up to individual NHS trusts. In line with the Diffusion of Innovation, guidelines could be adapted to meet the needs of individual trusts or hospitals.

The participants in this study did not mention organisational issues or cost effectiveness, which have been identified by Forsner et al. [16] as barriers to guideline implementation. This may suggest that while these barriers are important on an organisational issue they do not impact on daily prescribing.

### Compromise

The role of research is again highlighted in the final theme, which is the compromise between side effects and efficacy of antipsychotics. This issue has been discussed in multiple reviews including the CATIE and CUtLass trials [6, 8]. There is still much debate regarding the comparative efficacy of different antipsychotics, however there is a consensus on the side effects associated with particular antipsychotics. The participants who are aware of and discuss the adverse drug reactions appear to more risk averse. There is little evidence discussing the relationship between prescriber’s perception of risk and the choice of medication and whether this choice is based more heavily on the efficacy of the drugs or their risks.

### Strengths and limitations

This is the first study to qualitatively investigate the factors involved in clinical decision-making in the context of schizophrenia. These factors can be considered by organisations when implementing guidelines so that research evidence has a greater influence on clinical practice, ultimately improving patient care. Furthermore, it can help clinicians understand more about their decision-making process, thus allowing them to become more informed about their own clinical practice.

In this study data saturation was reached and all possible themes identified and explored. However, the participants were taken from the same organisation, which may have limited the themes identified and may reduce the generalisability of this research.

This study was conducted using thematic analysis. An alternative method, such as Interpretative Phenomenological Analysis, may have provided a more in-depth analysis by focusing further on individual participants.

### Future directions

The results from this research can help provide insight into some of the factors influencing clinical decision-making. Decision aids, such as those already used in for antidepressant prescribing created by the Mayo Clinic [28], may be created based on these findings to help healthcare professionals when planning treatment. Decision aids have been also been shown to increase patient’s knowledge [29], and recent evidence has shown that they are better when used by patients and clinician’s together to foster the shared decision making process [30] The decision aid is likely to be in a written or electronic form, with a potential for the role of pharmacogenetic decisions aids in the future as research into the role of these in mental health moves forward [31]. This is important in mental health where there are many treatment options and it is often difficult to know which one is best for the patient.

This study can be used to guide future research into prescribing behaviour. A Theory of Planned Behaviour study could be used to investigate these themes further and test the hypotheses created, for example the relationship between the perception of risk and the compromise between side effects and efficacy.

## Conclusion

It appears that clinicians use aspects of EBM to make clinical decisions. However, clinicians seem to place greater emphasis on clinical experience than previously thought. This implies that more decisions are made using system one of unconscious competence. The implications for clinical practice and for knowledge translation are that people should be encouraged to use more of the system two to understand their own behaviour and gain more insight into their decision-making behaviour. This could then be exploited when devising evidence implementation processes.

## Appendix

### Coding Framework

**Table:**

Code	Description
Information	Patient Presentation, clinical picture. Information from family, GP and other sources. Further information requested from or about the patient
Diagnosis	Differential diagnosis. Seeking facts to support Seeking test to support Are participants hesitant or confident about diagnosis
Plan	How it is proposed to manage patient
Risk	Risk to self or others. Risk of recurrence. Assessing whether it is worthwhile to take the risk Risk from medication
Side effects vs. Efficacy	Choice of drug – are side effects a factor? Effectiveness of treatment Side effects information Using side effects as a treatment strategy
Clinical Reasoning	Formulating arguments to support diagnosis or treatment plan
Clinical experience	To inform diagnosis or treatment Confidence or a lack of confidence
Drug Information	Describing the drug. Facts about its history or method of action.
Education and Training	Awareness of the source of their own knowledge and practices. Changes in practice following training and teaching
Evidence	Acknowledging evidence Assessing value and quantity of evidence available. Using evidence in their practice
Guidelines	Acknowledging current/changes in guidelines Using guidelines in their practice
Patient Involvement	Considering the patient’s ideas, concerns and expectations Including the patient in decision-making The influence of the patient on the participants practice Importance of the patient
Own beliefs	Opinions not based on specific information Opinions about other people’s beliefs

## Abbreviations

EBM: Evidence based medicine
NICE: National Institute of Clinical Excellence
